# Supplementary material for: Heterojunction formed via 3D-to-2D perovskite conversion for photostable wide-bandgap perovskite solar cells
Source: Nat Commun. 2023 Nov 6;14:7118. doi: 10.1038/s41467-023-43016-5 (PMC10628126; doi:10.1038/s41467-023-43016-5)
Supplement: Supplementary file 1 — Supplementary Information [file 41467_2023_43016_MOESM1_ESM.pdf]

Supplementary Information

for

Heterojunction formed via 3D-to-2D perovskite conversion for photostable wide-bandgap perovskite solar cells

Jin Wen[1], Yicheng Zhao[2], Pu Wu[1], Yuxuan Liu[1], Xuntian Zheng[1], Renxing Lin[1], Sushu Wan[3], Ke Li[3], Haowen Luo[1], Yuxi Tian[3], Ludong Li[1], and Hairen Tan[1]*

[1]*National Laboratory of Solid State Microstructures, Frontiers Science Center for Critical Earth Material Cycling, College of Engineering and Applied Sciences, Nanjing University, Nanjing 210023, China*

[2]*State Key Laboratory of Electronic Thin Films and Integrated Devices, School of Electronic Science and Engineering, University of Electronic Science and Technology of China, Chengdu 610054, China*

[3]*School of Chemistry and Chemical Engineering, Nanjing University, Nanjing 210023, China*

*Corresponding authors. E-mails: hairentan@nju.edu.cn

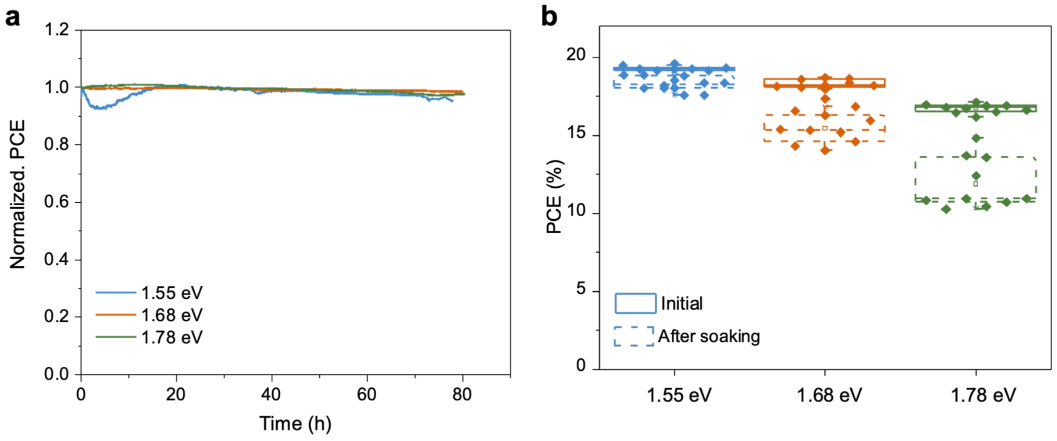

Supplementary Figure 1. Time evolution of efficiencies of 1.55, 1.68, and 1.78 eV devices measured at (**a**) maximum power point tracking (MPP) and (**b**) open-circuit (OC) conditions after 80 h under 1 sun illumination in ambient conditions (30% of relative humidity). The compositions are $Cs_{0.2}FA_{0.8}PbI_3$, $Cs_{0.2}FA_{0.8}Pb(I_{0.8}Br_{0.2})_3$ and $Cs_{0.2}FA_{0.8}Pb(I_{0.6}Br_{0.4})_3$. All devices were glass-encapsulated. The normalized efficiency is defined by the efficiency divided by the initial efficiency.

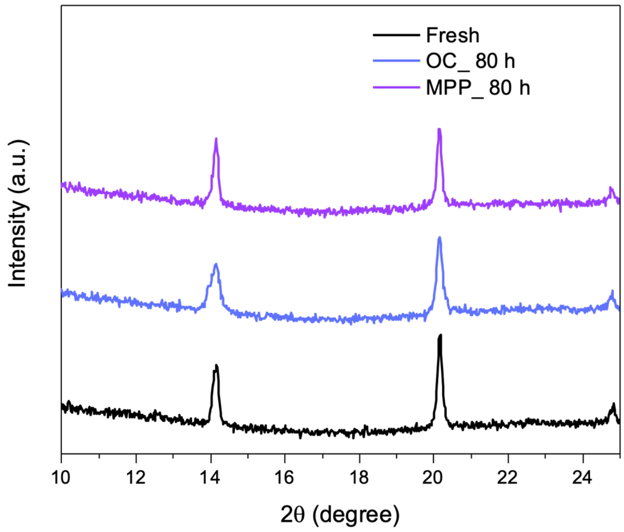

Supplementary Figure 2. XRD patterns of $Cs_{0.2}FA_{0.8}Pb(I_{0.6}Br_{0.4})_3$ perovskite devices before and after 80 h aging under MPP and OC conditions, respectively.

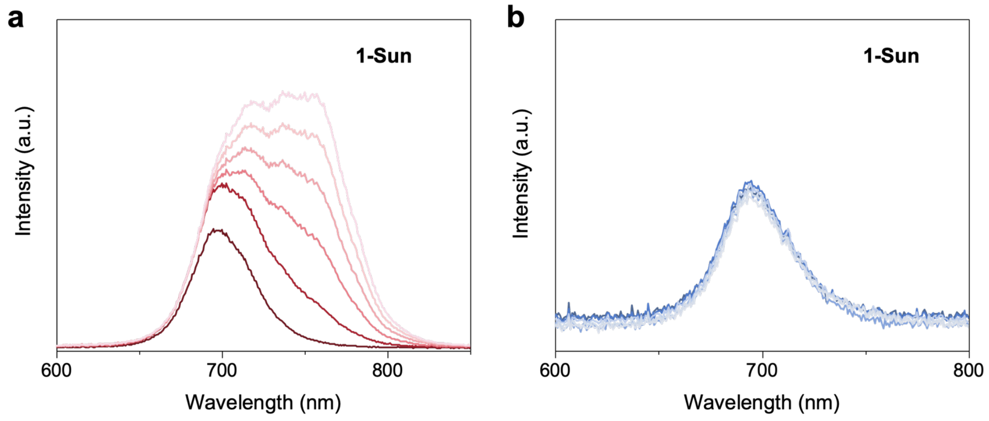

Supplementary Figure 3. PL spectra of perovskite device under illumination. **a** Open-circuit, **b** short-circuit conditions. The samples were excited under a 532 nm laser for 60 min.

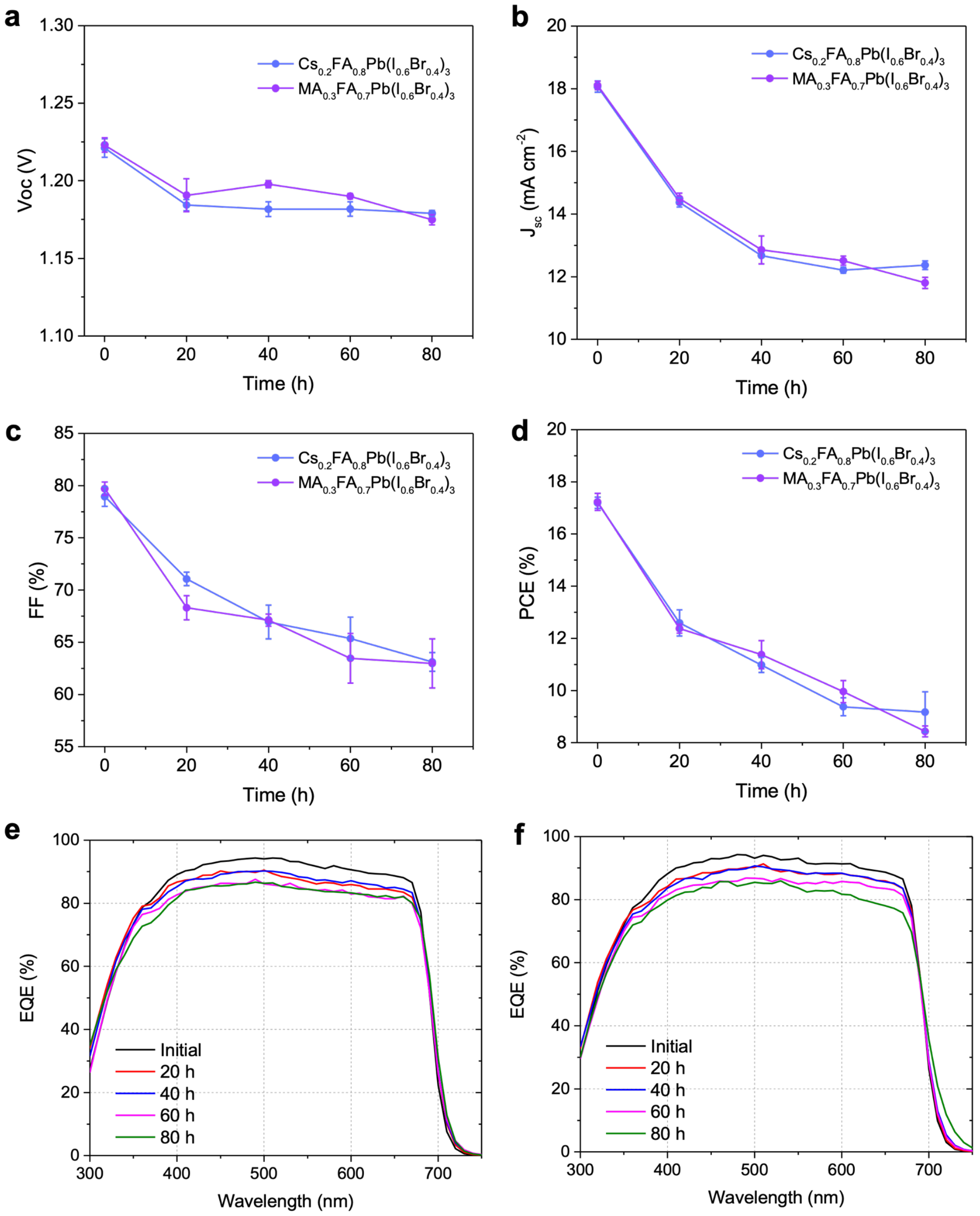

Supplementary Figure 4. a-d Evolution of PV parameters of WBG perovskite solar cells under simulated one sun illumination with FACs/FAMA compositions. **e-f** EQE evolution of $Cs_{0.2}FA_{0.8}Pb(I_{0.6}Br_{0.4})_3$ and $MA_{0.3}FA_{0.7}Pb(I_{0.6}Br_{0.4})_3$ devices under simulated one sun illumination.

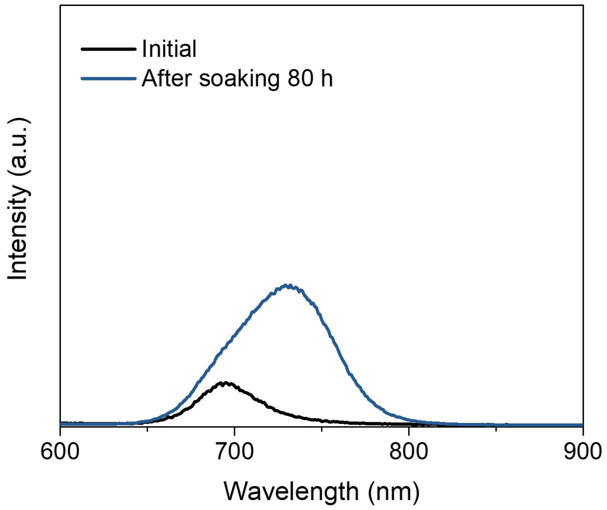

Supplementary Figure 5. PL spectra of $Cs_{0.2}FA_{0.8}Pb(I_{0.6}Br_{0.4})_3$ perovskite device before and after light soaking.

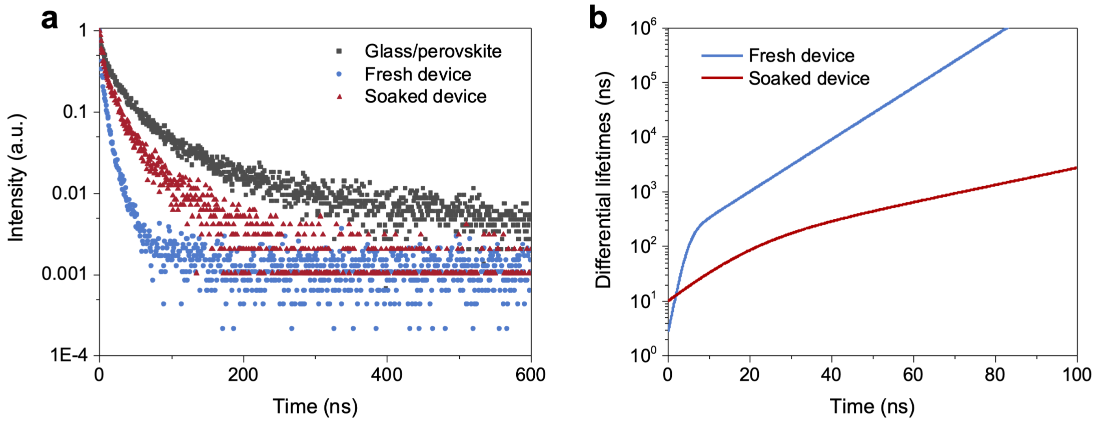

Supplementary Figure 6. a TRPL spectra of $Cs_{0.2}FA_{0.8}Pb(I_{0.6}Br_{0.4})_3$ perovskite device before and after light soaking. **b** Computed differential lifetimes obtained by taking the derivative from fits to the transients in (a).

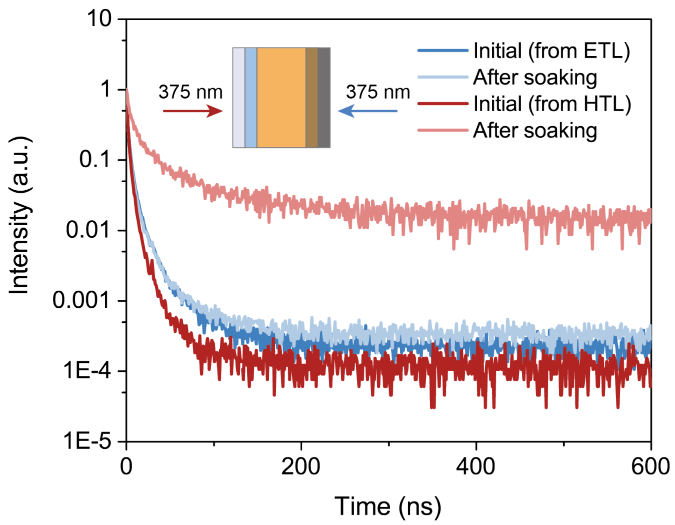

Supplementary Figure 7. TRPL spectra of perovskite devices excited by a 375 nm laser from the C_{60} side and from the NiO_x/SAM side before and after light soaking, respectively.

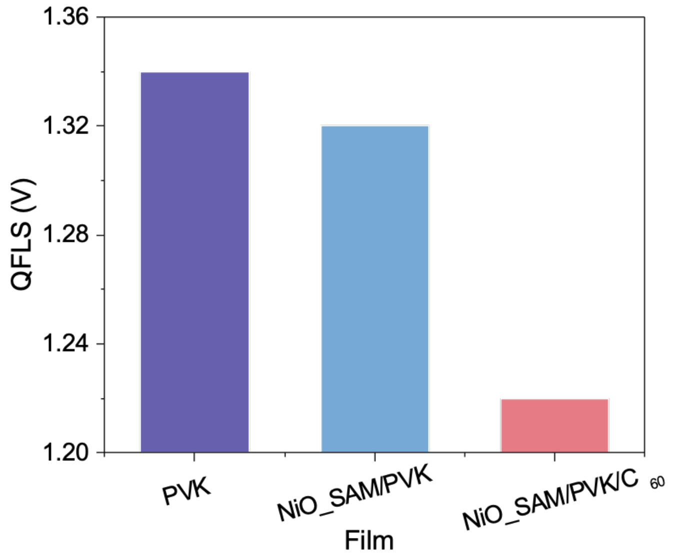

Supplementary Figure 8. The calculated QFLS of the perovskite film, HTL/perovskite and HTL/perovskite/electron transport layer junctions.

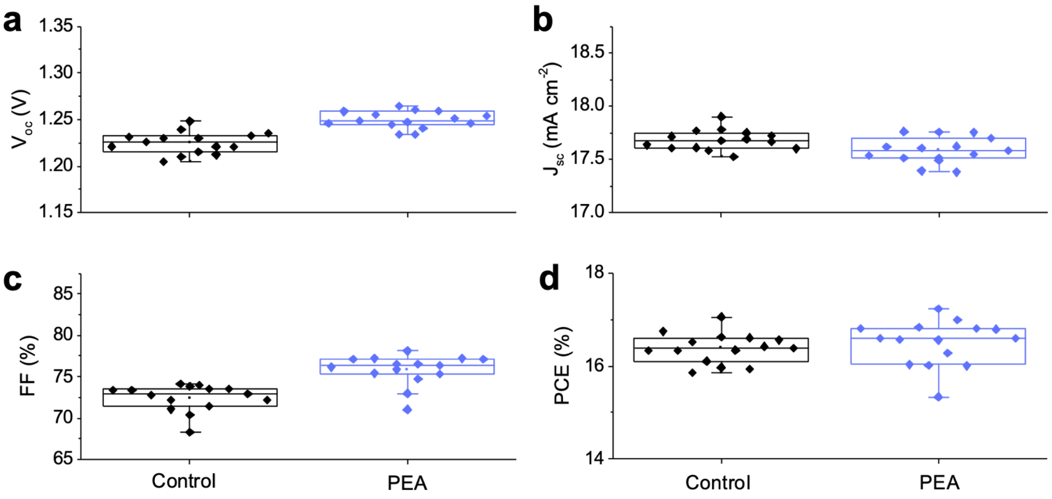

Supplementary Figure 9. PV parameters of devices with or without PEAI passivation (15 devices for each type). **a** V_{oc}, **b** J_{sc}, **c** FF, and **d** PCE.

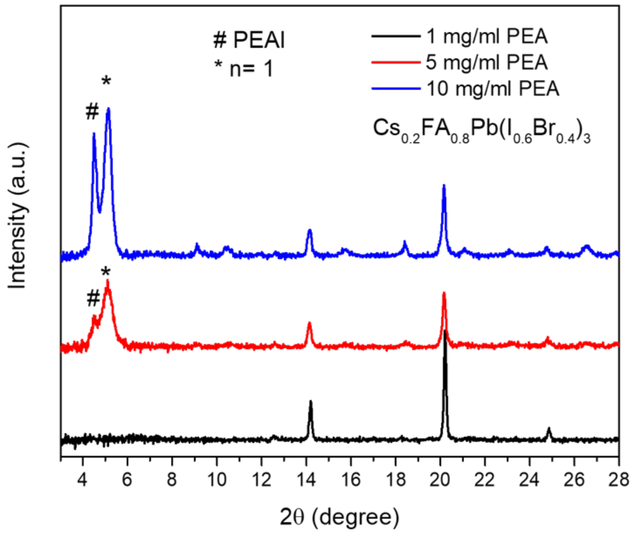

Supplementary Figure 10. XRD patterns of $Cs_{0.2}FA_{0.8}Pb(I_{0.6}Br_{0.4})_3$ with 1, 5 and 10 mg/ml PEAI treatment. The application of PEAI solutions at various concentrations aimed to overcome the detection limitations for 2D structures formed at lower concentrations (1 mg mL^{-1}) by XRD testing, while higher concentrations (5 and 10 mg mL^{-1}) demonstrated the exclusive formation of n = 1 2D structures on the WBG perovskite surface.

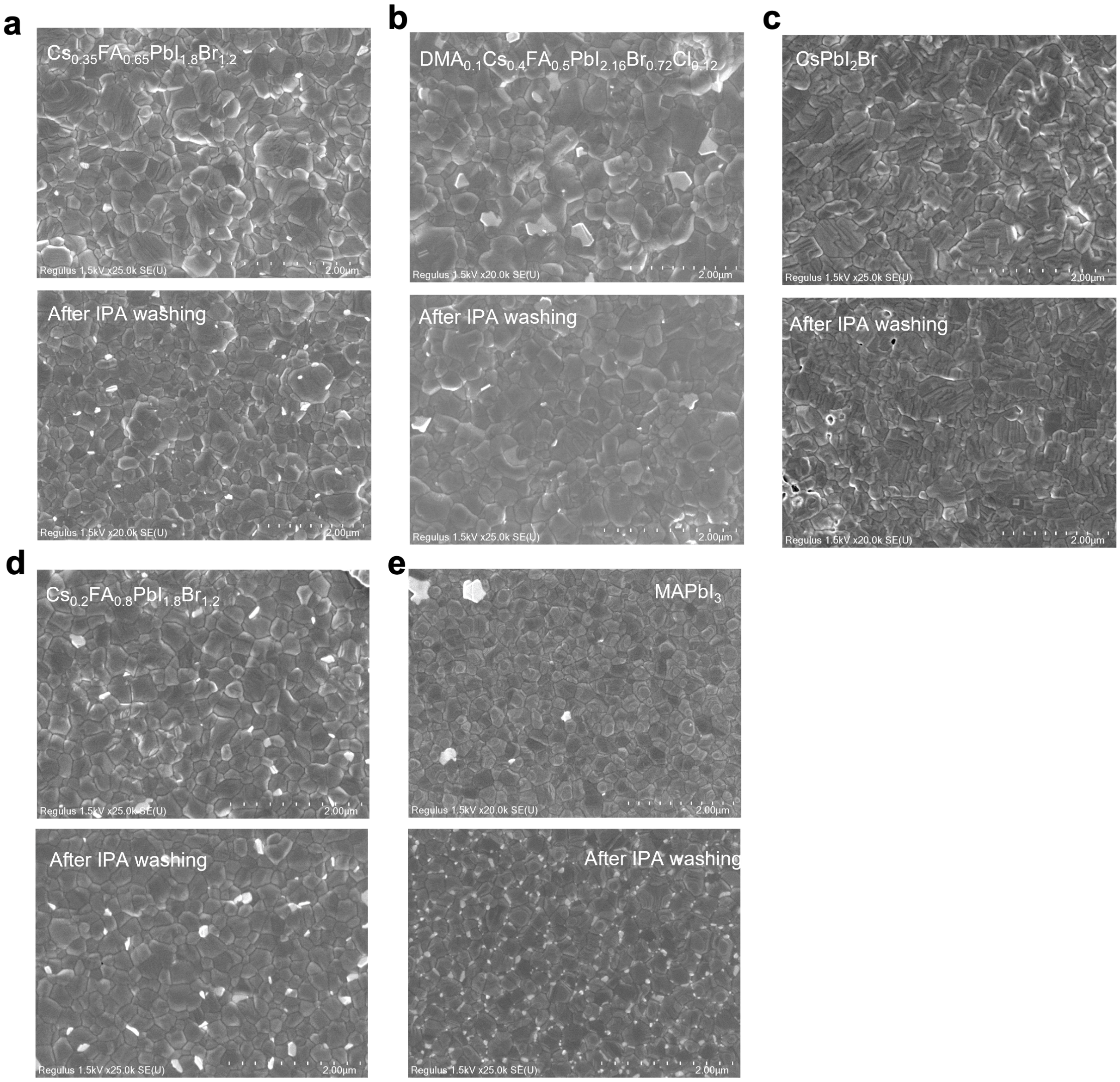

Supplementary Figure 11. SEM images of (**a**) $Cs_{0.35}FA_{0.65}PbI_{1.8}Br_{1.2}$, (**b**) $DMA_{0.1}Cs_{0.4}FA_{0.5}PbI_{2.16}Br_{0.72}Cl_{0.12}$, (**c**) $CsPbI_2Br$, (**d**) $Cs_{0.2}FA_{0.8}PbI_{1.8}Br_{1.2}$ and (**e**) $MAPbI_3$ films with different compositions before and after IPA washing.

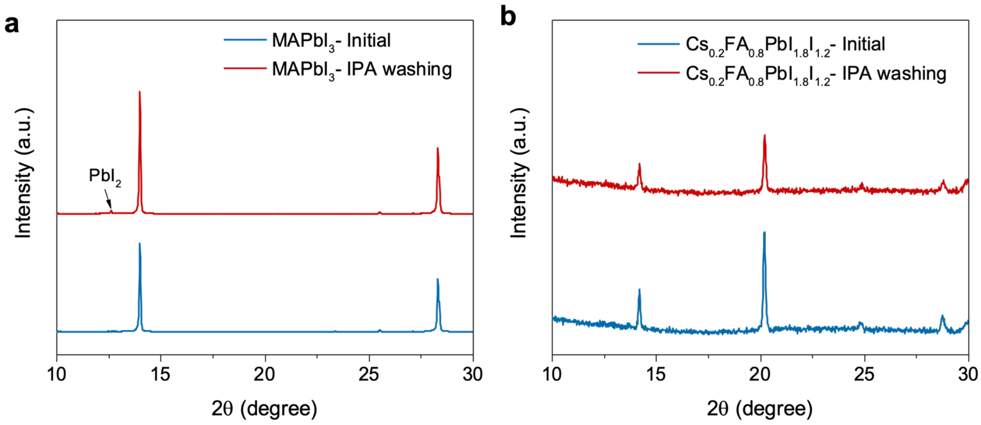

Supplementary Figure 12. XRD pattern of (**a**) $MAPbI_3$ and (**b**) $Cs_{0.2}FA_{0.8}PbI_{1.8}Br_{1.2}$ perovskite films before and after IPA washing.

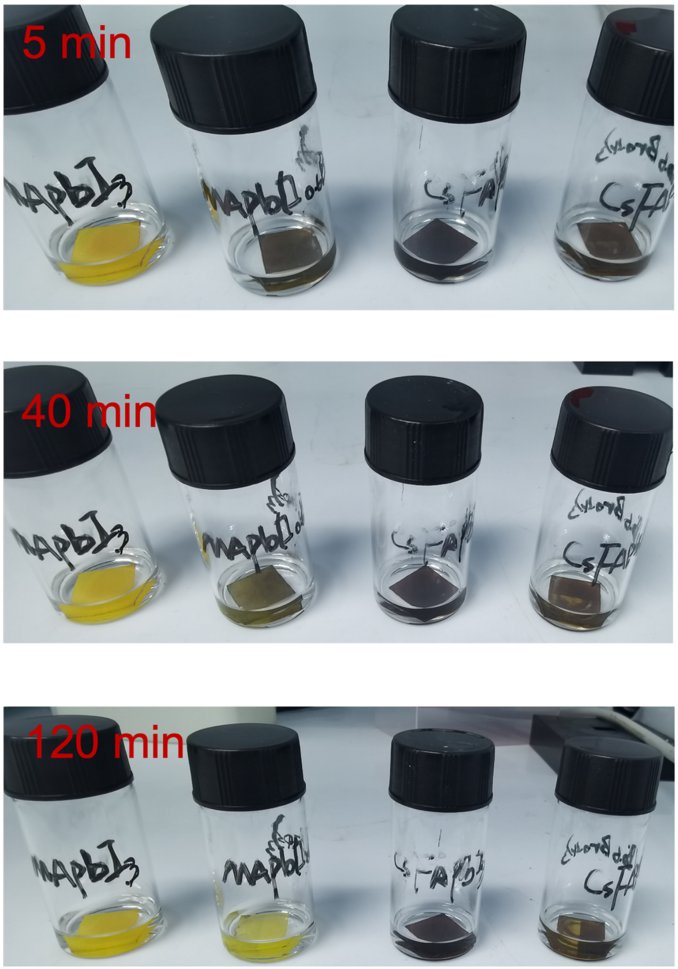

Supplementary Figure 13. Photos of films of $MAPbI_3$, $MAPb(I_{0.6}Br_{0.4})_3$, $Cs_{0.2}FA_{0.8}PbI_3$, and $Cs_{0.2}FA_{0.8}Pb(I_{0.6}Br_{0.4})_3$ (from left to right) soaked in the IPA.

Supplementary Note 1

To achieve a better effect of improving the performance of the WBG PSC with a quasi-2D layer, we have optimized the processing of the VAQ-2D layer. We observed that PbI_2 layers thinner than 10 nm fail to form a continuous film on the surface of the 3D perovskite. To investigate the relationship between MAI concentration and the conversion of PbI_2 thickness to $MAPbI_3$, we conducted depositions of 10 nm and 20 nm PbI_2 on a glass substrate, as illustrated in Supplementary Fig. 15. Our findings revealed that MAI concentrations exceeding 2 mg mL^{-1}can entirely convert 10 nm thick PbI_2, while concentrations exceeding 4 mg mL^{-1} are necessary for full conversion of 20 nm thick PbI_2. Corresponding SEM images in Supplementary Fig. 16 depict the transformation of 2 mg mL^{-1} MAI with 10 nm thick PbI_2 into well-defined crystalline grains, whereas higher concentrations result in the formation of amorphous thin layers.

Given the inherent challenges in accurately characterizing thin 2D layers, we determined the optimal PEAI concentration based on device performance. As demonstrated in Supplementary Figure 17-18, the peak device performance is achieved with a combination of 10 nm thick PbI_2, 2 mg mL^{-1} MAI, and 2 mg mL^{-1} PEAI. Nevertheless, when the PbI_2 thickness is increased to 20 nm, a discernible reduction in both J_{sc} and FF is observed. This decline is attributed to the excessive thickness of the 2D layer, which hampers charge transport at the interface. Thus, we selected the combination of 10 nm thick PbI_2, 2 mg mL^{-1} MAI, and 2 mg mL^{-1} PEAI for the champion device.

While the diffraction peaks of PbI_2 and $MAPbI_3$ were detected during the optimized processing, the XRD pattern and PL emission from the resulting ultra-thin 2D perovskite layer were not observed (Supplementary Fig. 19). However, when the same processing was applied to deposit the 2D layer on glass, the XRD peak of VAQ-2D was detected, confirming the formation of 2D perovskite layers with $n \geq 2$ (Supplementary Fig. 19). Additionally, when the PbI_2 layer was thickened to 20 nm, PL emission peaks at 560, 610, and 650 nm (corresponding to $n = 2$, 3, and 4, respectively) were observed in the films, providing further confirmation of the VAQ-2D structure.

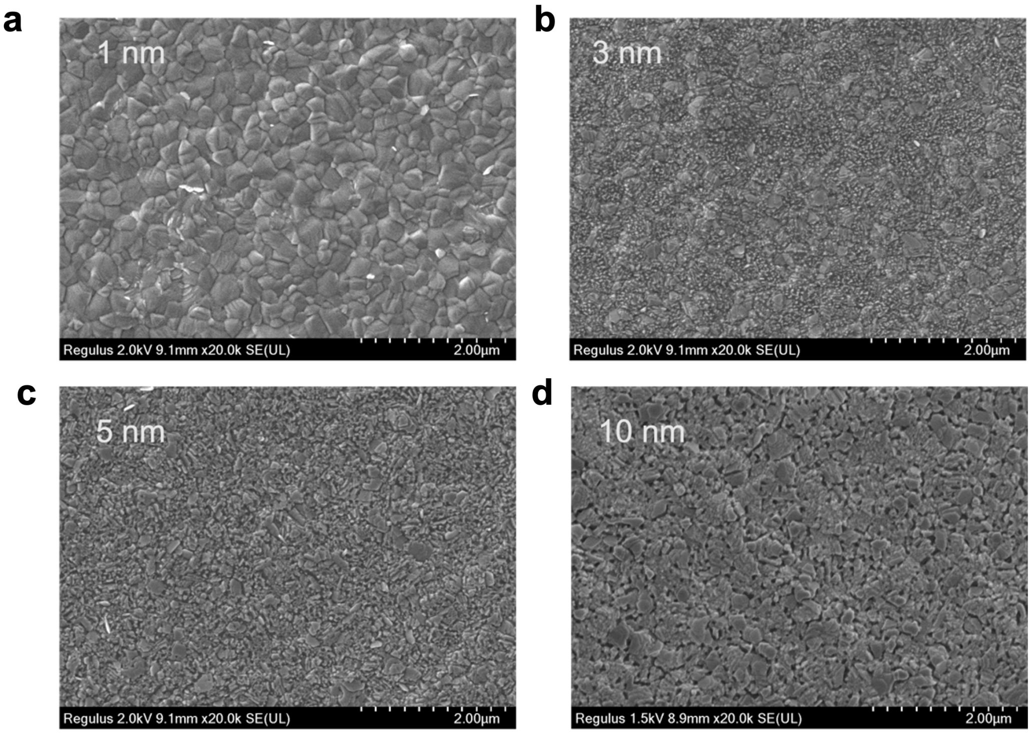

Supplementary Figure 14. Top-view SEM image of WBG perovskite films with various PbI_2 thicknesses. We mark the thickness of the PbI_2 from the evaporation equipment (**a**) 1nm, (**b**) 3 nm, (**c**) 5 nm and (**d**) 10 nm.

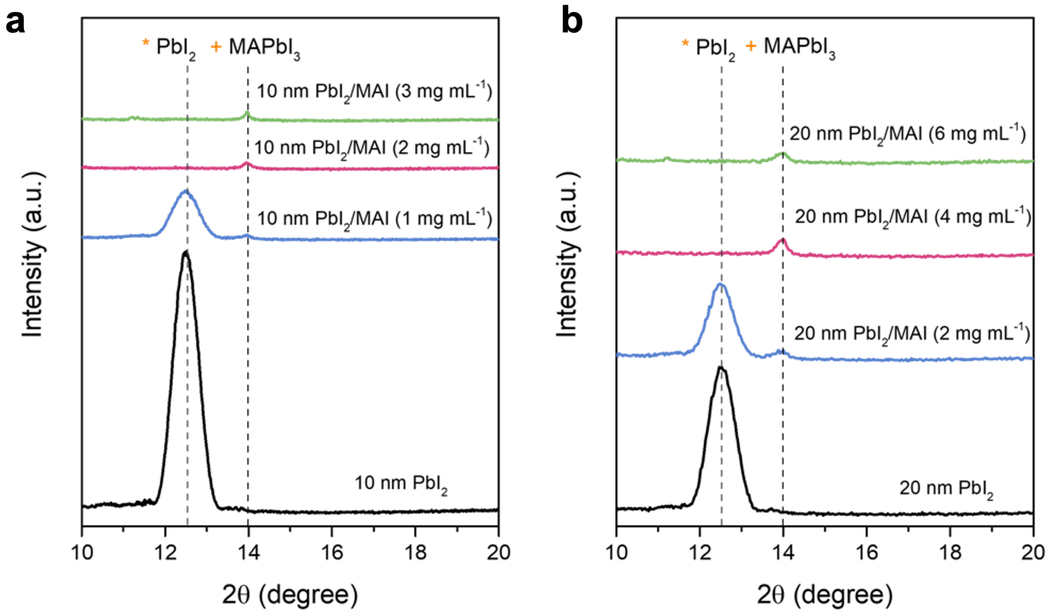

Supplementary Figure 15. XRD patterns of (**a**) 10 nm PbI_2 film and (**b**) 20 nm PbI_2 film deposited on the glass substrate and converted by MAI solution with various concentrations.

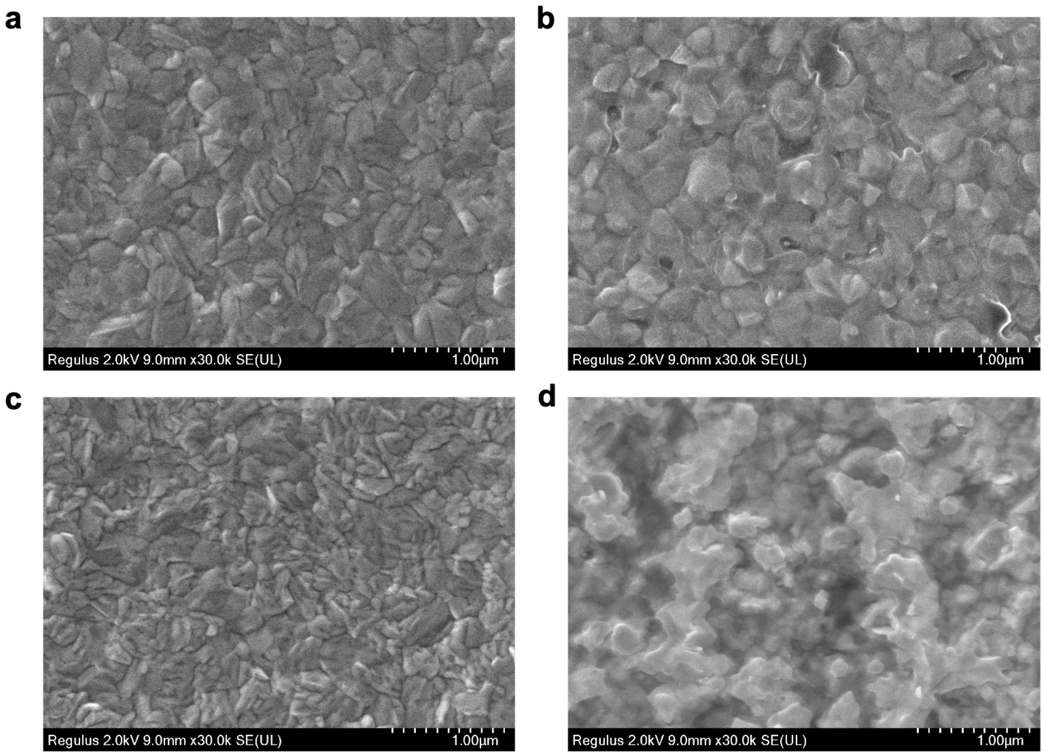

Supplementary Figure 16. Top-view SEM image of PbI_2 films deposited upon WBG perovskite film and reacting with various contents of MAI in the organic salt. **a** PbI_2 (10 nm) /MAI (2 mg mL^{-1}), **b** PbI_2 (10 nm) /MAI (3 mg mL^{-1}), **c** PbI_2 (20 nm) /MAI (4 mg mL^{-1}) and **d** PbI_2 (20 nm) /MAI (6 mg mL^{-1}).

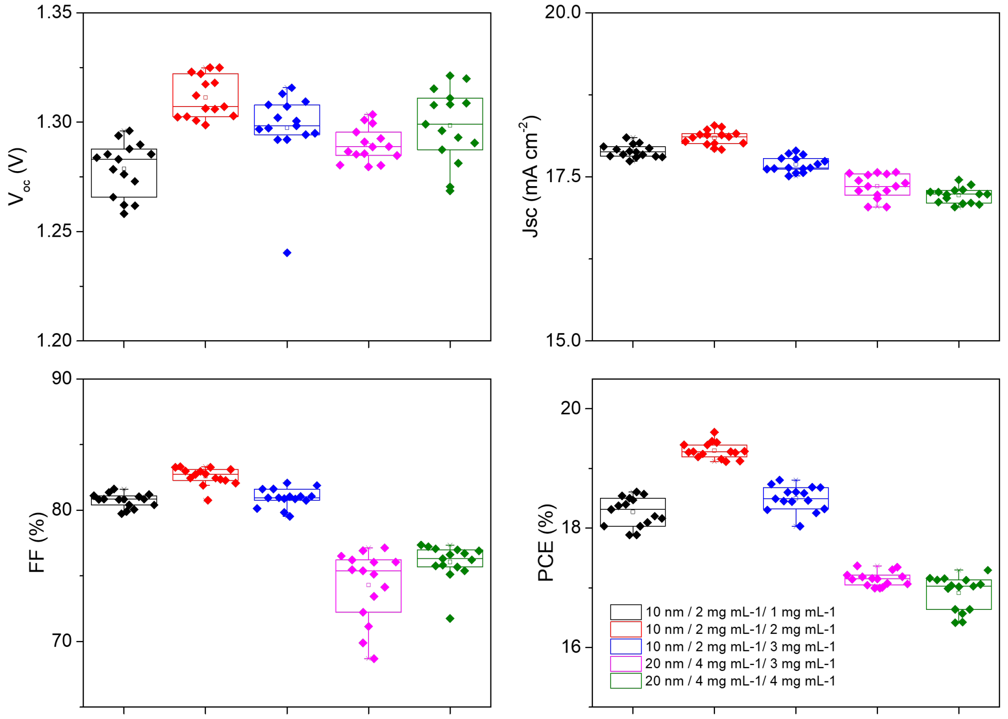

Supplementary Figure 17. PV parameters of devices with different combinations of PbI_2, MAI and PEAI.

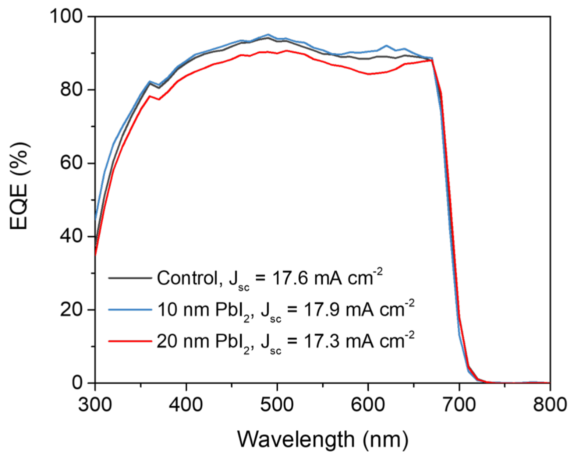

Supplementary Figure 18. EQE curves of WBG devices treated with VAQ-2D of varying thicknesses.

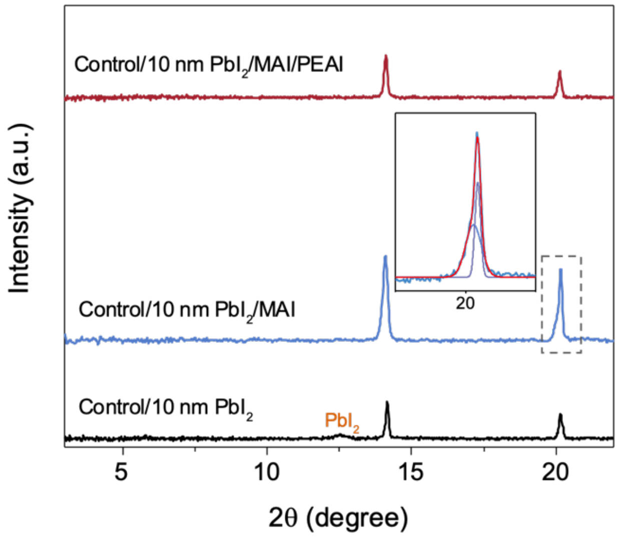

Supplementary Figure 19. XRD patterns of WBG perovskite during the VAQ-2D deposition.

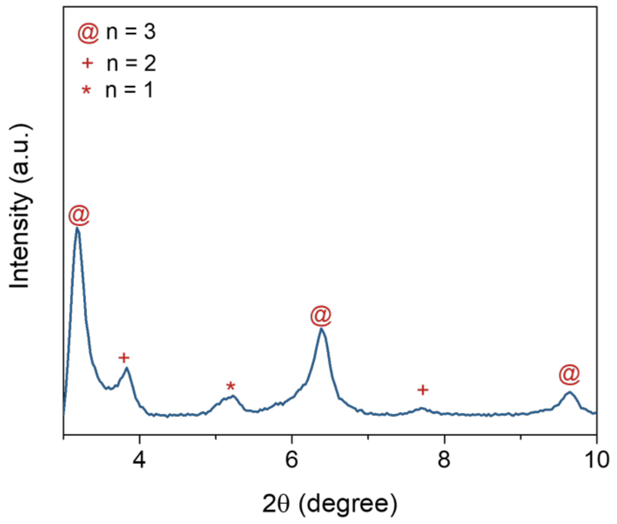

Supplementary Figure 20. XRD pattern of quasi-2D perovskite fabricated on the glass by vapor-assisted strategy.

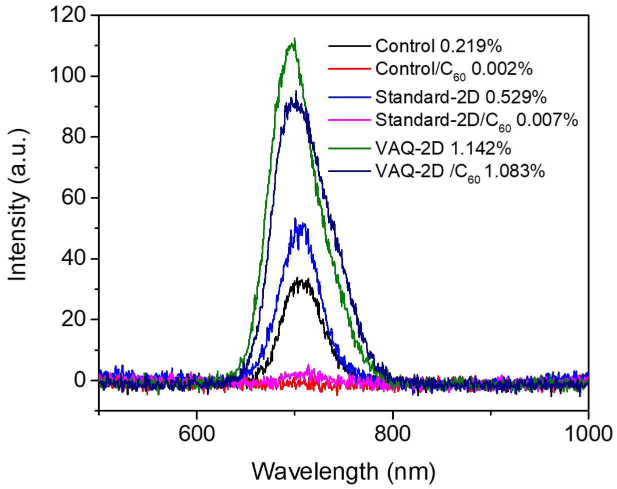

Supplementary Figure 21. PLQY of the perovskite films, HTL/perovskite and HTL/perovskite/electron transport layer junctions.

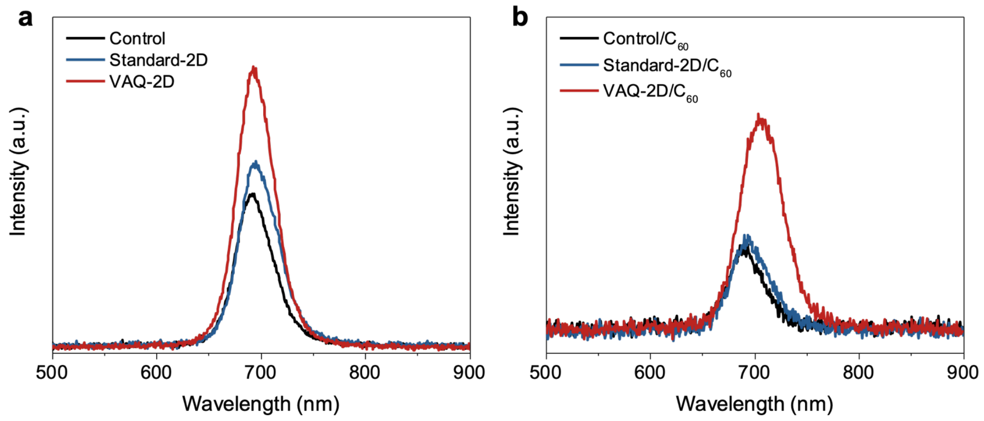

Supplementary Figure 22. Steady-state PL spectra of perovskite films (**a**) without and (**b**) with C_{60} coated.

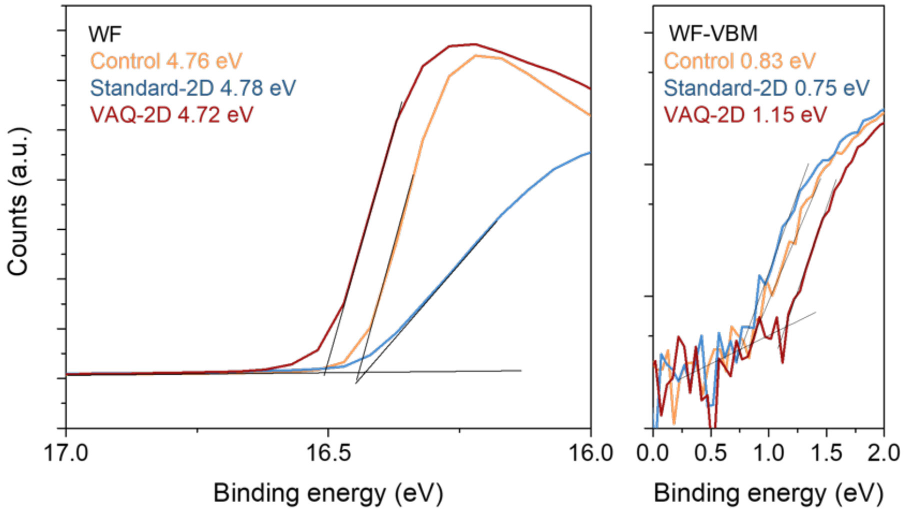

Supplementary Figure 23. UPS spectra in the valence band (VB) region and secondary electron cut-off plots for control, standard-2D and quasi-2D films.

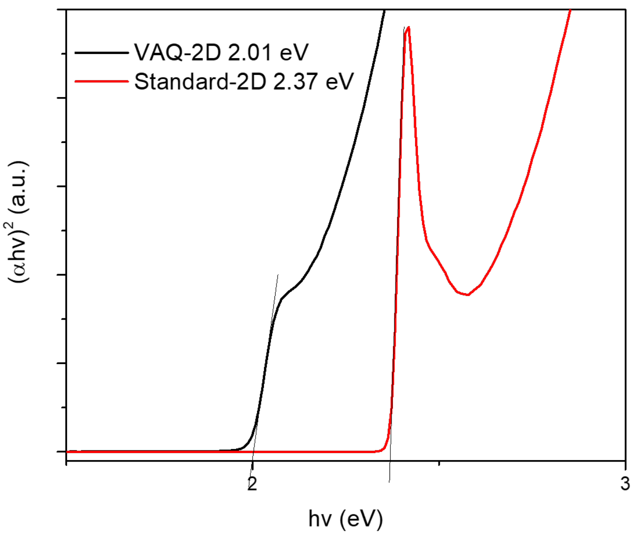

Supplementary Figure 24. Tauc plots of 2D perovskite films deposited on glass.

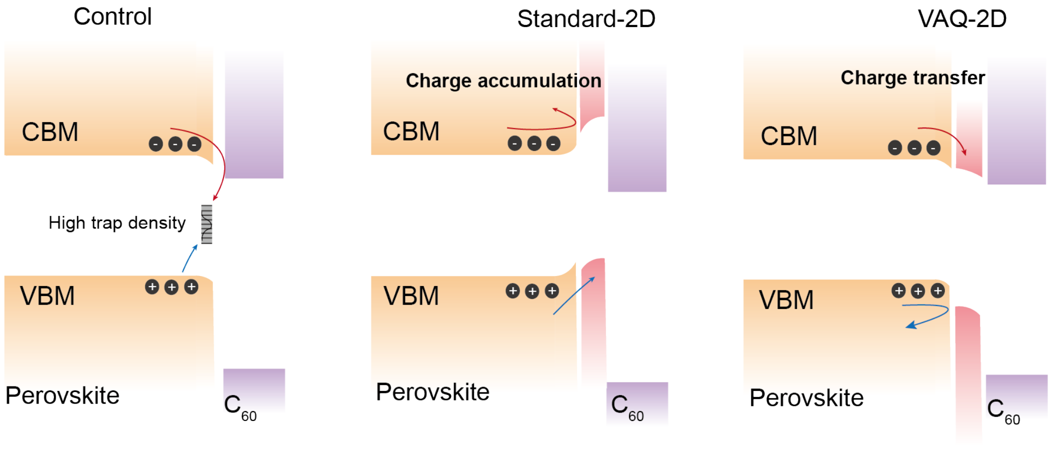

Supplementary Figure 25. Schematic of energy diagram for the control standard-2D and VAQ-2D perovskite solar cells. The red and blue lines denote the non-radiative recombination pathways and the directions of carrier drift.

Table S1. Fitting results of time-resolved photoluminescence spectra. The effective carrier lifetime was calculated using biexponential fitting $Y = A_1 exp(-t/t_1) + A_2 exp(-t/t_2)$, where A_1 and A_2 are the relative amplitudes, and t_1 and t_2 are the lifetimes for the fast and slow recombination, respectively.

**Sample**	**t_1 (ns)**	**t_2 (ns)**
Control	4.5	30.3
Standard-2D	7.1	74.5
Quasi-2D	16.3	177.6
Control/C_{60}	3.3	14.0
Standard-2D/C_{60}	4.0	18.1
Quasi-2D/C_{60}	1.7	31.2

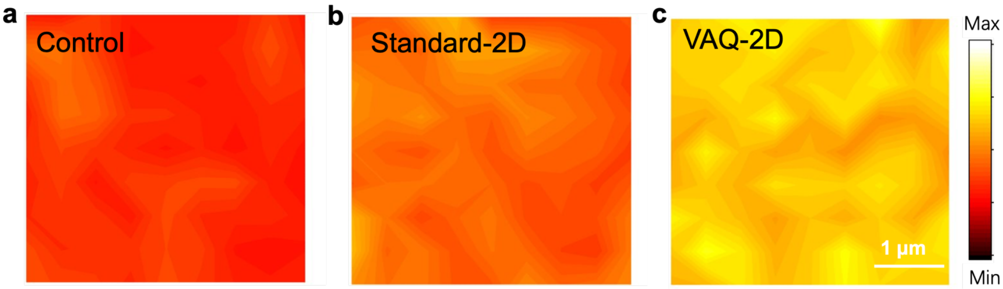

Supplementary Figure 26. PL mappings of (**a**) ITO/NiO_x/SAM/perovskite/C_{60}, (**b**) ITO/NiO_x/SAM/perovskite/standard-2D/C_{60}, and (**c**) ITO/ NiO_x/SAM /perovskite/VAQ-2D/C_{60} excited from the C_{60} side with a 485-nm laser.

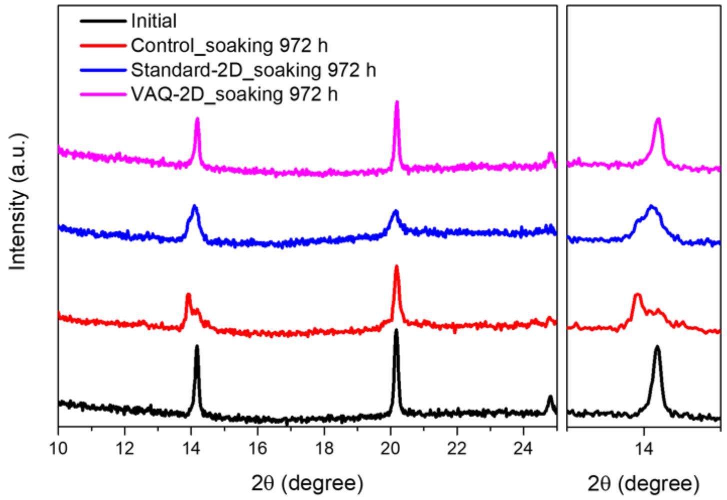

Supplementary Figure 27. XRD patterns of the initial device and the device of control, standard-2D, VAQ-2D aged after aging of 972 h under illumination.

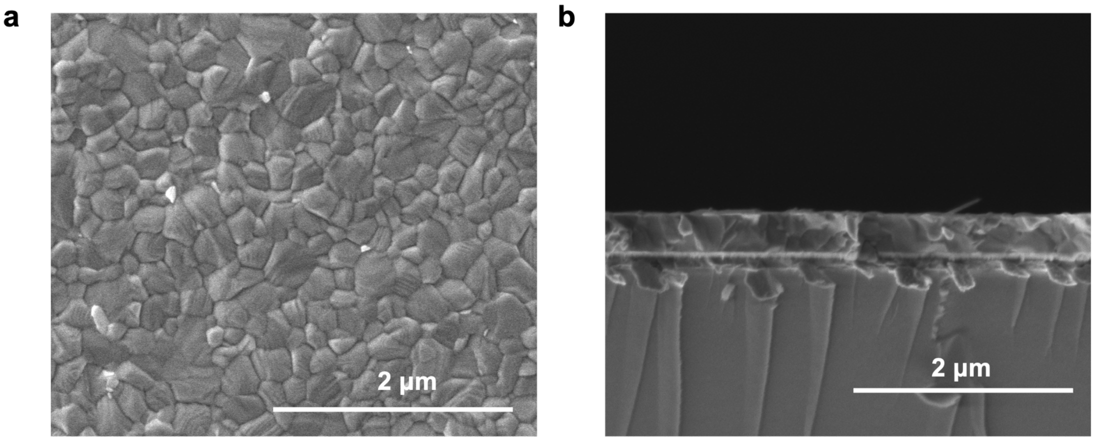

Supplementary Figure 28. a Top-view SEM image of as-fabricated WBG perovskite film. **b** Cross-sectional SEM images of as-fabricated PSCs.

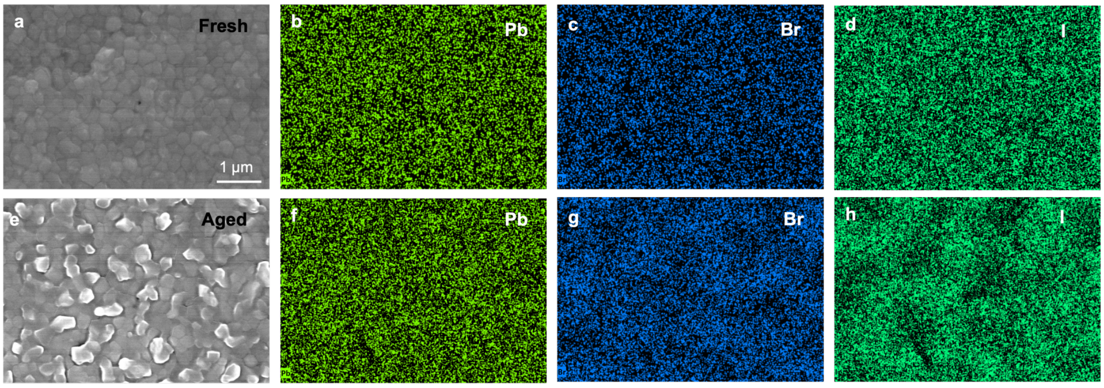

Supplementary Figure 29. SEM and EDS mapping of VAQ-2D film (**a-d**) before and (**e-h**) after soaking 500 h.

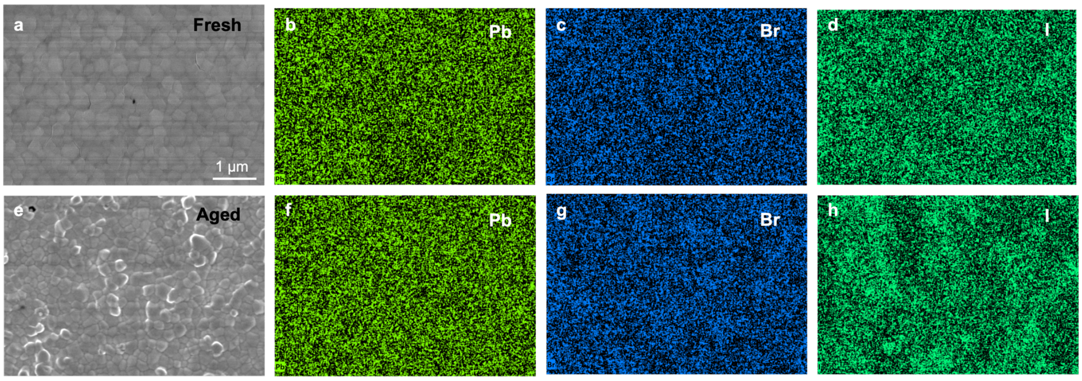

Supplementary Figure 30. SEM and EDS mapping of VAQ-2D film (**a-d**) before and (**e-h**) after soaking 500 h.

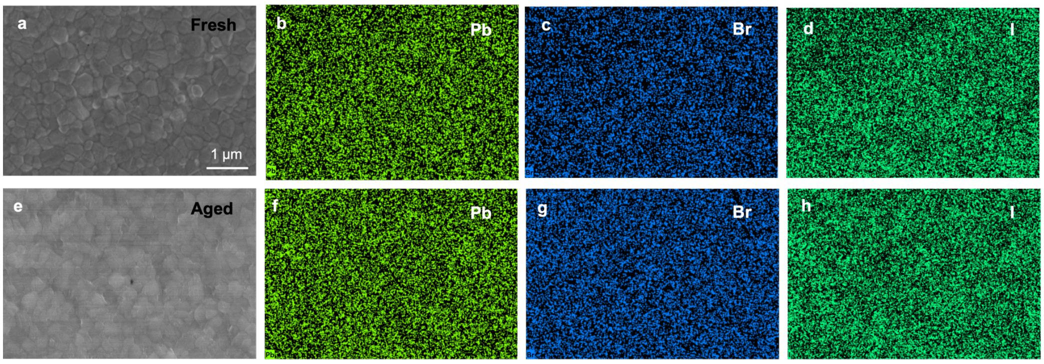

Supplementary Figure 31. SEM and EDS mapping of VAQ-2D film (**a-d**) before and (**e-h**) after soaking 500 h.

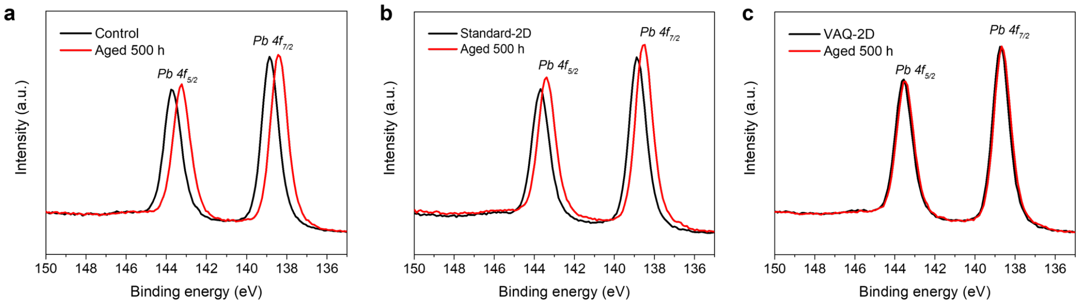

Supplementary Figure 32. XPS spectra of *Pb 4f* for (**a**) control, (**b**) standard-2D and (**c**) VAQ-2D before and after aging.

Supplementary Note 2

In the absence of the MAI step, devices with a deposited layer of PbI_2 followed by PEAI treatment displayed a more pronounced reduction in current compared to those directly spin-coated with PEAI (Supplementary Fig. 33). This effect can be attributed to the thicker n=1 2D layer impeding charge transport, emphasizing the essential role of MA^+ in the formation of higher n-value 2D structures.

Supplementary Fig. 34 illustrates the evolution of device performance during the three-step fabrication of VAQ-2D. Following the deposition of PbI_2, the device performance shows an increase in V_{oc} and FF. However, due to the wider energy levels impeding charge transport, the J_{sc} exhibits a significant decrease. After MAI treatment, the surface transitions to the narrower energy levels of $MAPbI_3$, resulting in an increase in J_{sc} but decreased V_{oc} and FF. Finally, after PEAI treatment, the surface achieves an optimal energy level, resulting in a substantial enhancement in all three device parameters.

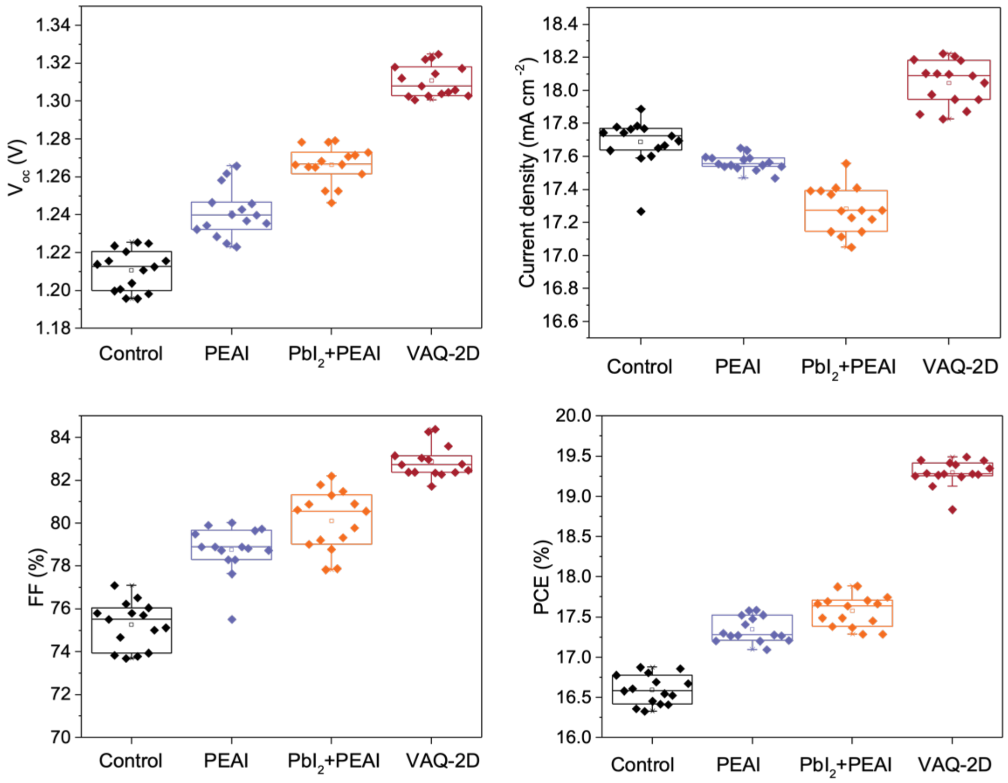

Supplementary Figure 33. PV parameters of devices with different passivating methods (15 devices for each type): PEAI solution, vapored-PbI_2 + PEAI solution, vapored-PbI_2 + PEAI solution, vapored-PbI_2 + MAI + PEAI solution.

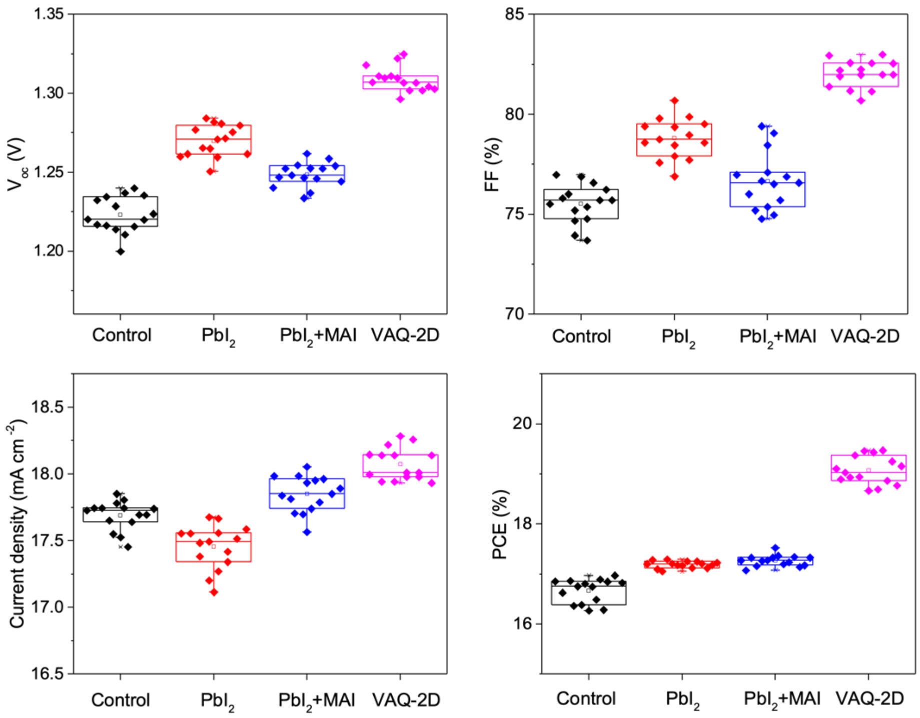

Supplementary Figure 34. Performance of WBG perovskite solar cells during vapor-assisted 3D-to-2D conversion method. Statistical performance of 15 devices for each type is presented. The optimal thickness of the evaporated PbI_2 layer is 10 nm, which is subsequently transformed into a $MAPbI_3$ perovskite layer after organic-salt (MAI) treatment. Finally, it is converted into a quasi-two-dimensional structure through PEAI treatment.

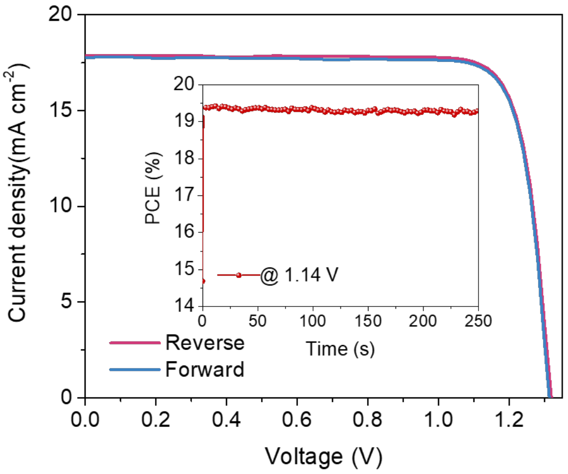

Supplementary Figure 35. *J-V* curves of the champion quasi-2D device. The inset shows the steady-state output of the champion device for 250 s, exhibiting a stabilized PCE of 19.6%.

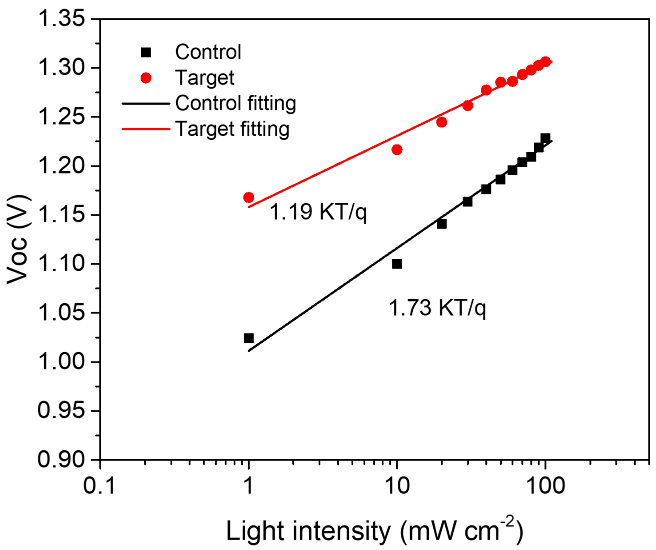

Supplementary Figure 36. The V_{oc} of reference and target devices were plotted as a function of light intensity.

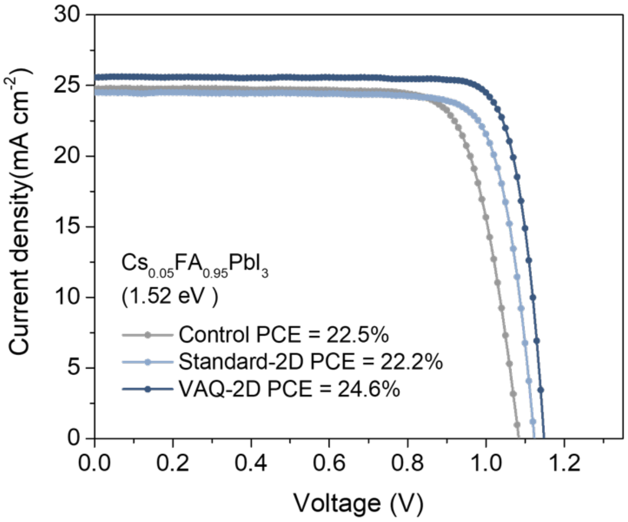

Supplementary Figure 37. *J-V* curves of the control, standard-2D and quasi-2D devices for $Cs_{0.05}FA_{0.95}I_3$.

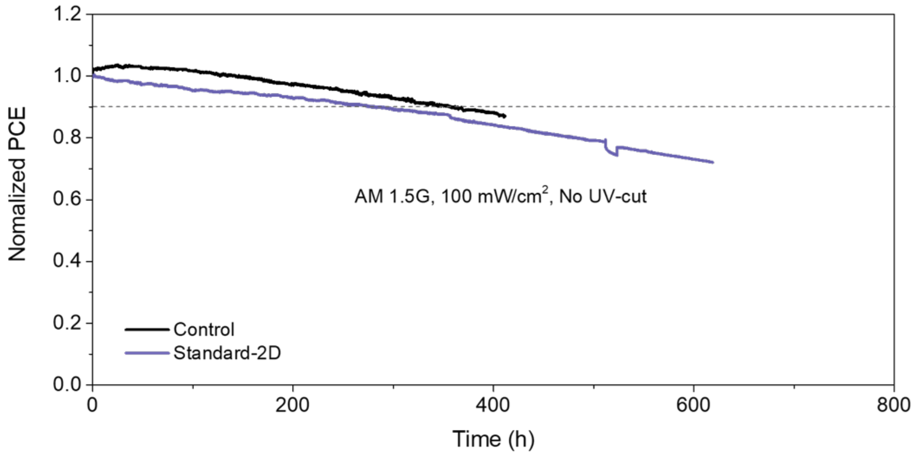

Supplementary Figure 38. MPP tracking was measured with the encapsulated control and standard-2D devices under full solar illumination (AM 1.5G, 100 mW cm^{-2}) in ambient conditions.

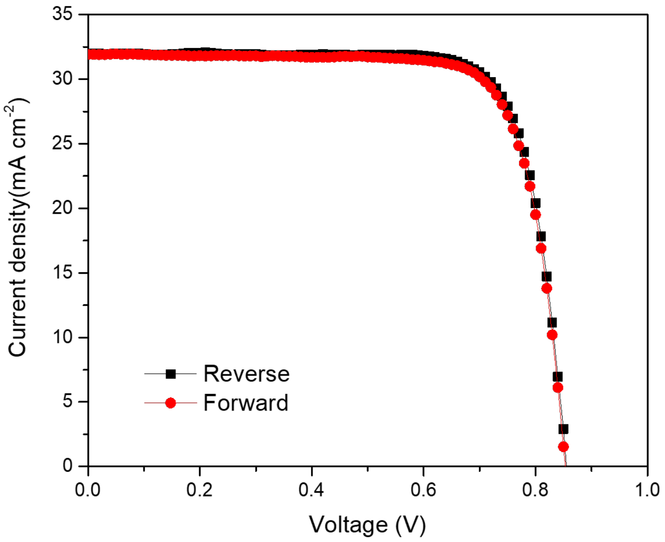

Supplementary Figure 39. *J-V* curves of the narrow-bandgap single-junction perovskite solar cell, show PCEs of 21.6% and 21.3% under reverse and forward scans, respectively.

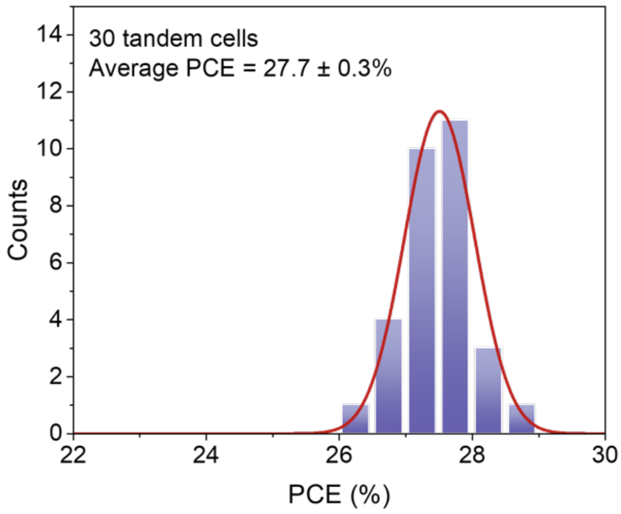

Supplementary Figure 40. The PCE histogram of 30 tandem devices, exhibits an average PCE of 27.7 ± 0.3%.

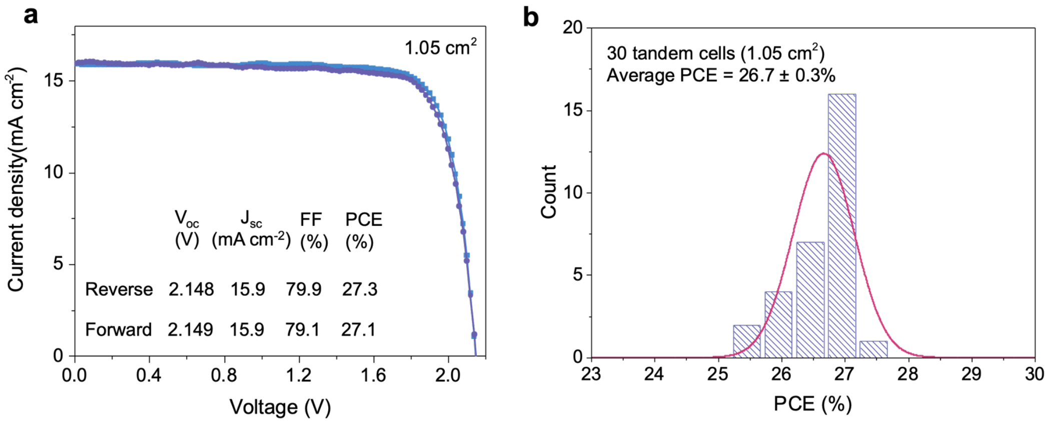

Supplementary Figure 41. a *J-V* curves of the champion tandem cell with an aperture area of 1.05 cm^2. **b** PCE distribution of 30 tandem devices (1.05 cm^2), showing an average PCE of 26.7± 0.3%.

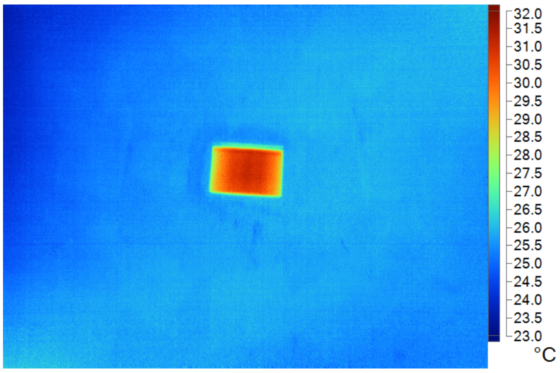

Supplementary Figure 42. The temperature of the tandem device under MPP operation, measured by an infrared camera.

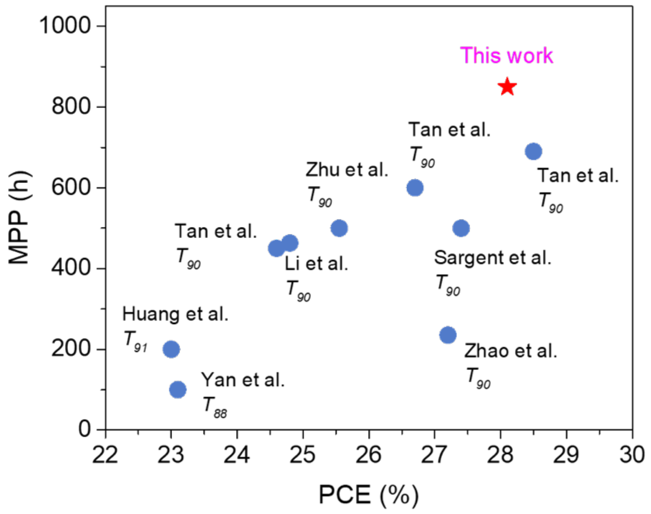

Supplementary Figure 43. Reported PCE versus operation stability values for all-perovskite tandem solar cells[1–9]. The red star represents this work.

Supplementary References

1. Zhao, D. *et al.* Efficient two-terminal all-perovskite tandem solar cells enabled by high-quality low-bandgap absorber layers. *Nat. Energy* **3**, 1093–1100 (2018).
2. Tong, J. *et al.* Carrier lifetimes of >1 ms in Sn-Pb perovskites enable efficient all-perovskite tandem solar cells. *Science.* **364**, 475–479 (2019).
3. Lin, R. *et al.* Monolithic all-perovskite tandem solar cells with 24.8% efficiency exploiting comproportionation to suppress Sn(ii) oxidation in precursor ink. *Nat. Energy* **4**, 864–873 (2019).
4. Yang, Z. *et al.* Enhancing electron diffusion length in narrow-bandgap perovskites for efficient monolithic perovskite tandem solar cells. *Nat. Commun.* **10**, 4498 (2019).
5. Lin, R. *et al.* All-perovskite tandem solar cells with improved grain surface passivation. *Nature* **603**, 73–78 (2022).
6. Zhu, J. *et al.* A donor–acceptor-type hole-selective contact reducing non-radiative recombination losses in both subcells towards efficient all-perovskite tandems. *Nat Energy* **8**, 714–724 (2023).
7. Jiang, Q. *et al.* Compositional texture engineering for highly stable wide-bandgap perovskite solar cells. *Science* **378**, 1295–1300 (2022).
8. Lin, R. *et al.* All-perovskite tandem solar cells with 3D/3D bilayer perovskite heterojunction. *Nature* **620**, 994–1000 (2023).
9. Chen, H. et al. Regulating surface potential maximizes voltage in all-perovskite tandems. Nature 613, 676–681 (2022).
